# On the First Quantum Correction to the Second Virial Coefficient of a Generalized Lennard-Jones Fluid

**DOI:** 10.3390/e27121251

**Published:** 2025-12-11

**Authors:** Daniel Parejo, Andrés Santos

**Affiliations:** 1 Departamento de Física, Universidad de Extremadura, E-06006 Badajoz, Spain; dparejof@alumnos.unex.es; 2 Instituto de Computación Científica Avanzada (ICCAEx), Universidad de Extremadura, E-06006 Badajoz, Spain

**Keywords:** second virial coefficient, quantum corrections, generalized Lennard-Jones potential, semiclassical fluids, parabolic cylinder functions

## Abstract

We derive an explicit analytic expression for the first quantum correction to the second virial coefficient of a *d*-dimensional fluid whose particles interact via the generalized Lennard-Jones (2n,n) potential. By introducing an appropriate change of variable, the correction term is reduced to a single integral that can be evaluated in closed form in terms of parabolic cylinder or generalized Hermite functions. The resulting expression compactly incorporates both dimensionality and stiffness, providing direct access to the low- and high-temperature asymptotic regimes. In the special case of the standard Lennard-Jones fluid (d=3, n=6), the formula obtained is considerably more compact than previously reported representations based on hypergeometric functions. The knowledge of this correction allows us to determine the first quantum contribution to the Boyle temperature, whose dependence on dimensionality and stiffness is explicitly analyzed, and enables quantitative assessment of quantum effects in noble gases such as helium, neon, and argon. Moreover, the same methodology can be systematically extended to obtain higher-order quantum corrections.

## 1. Introduction

In a semiclassical fluid, the second virial coefficient can be expressed as follows [1,2,3,4,5,6,7,8]:

(1) $$B_{2}\left(T\right)=B^{\left(0\right)}\left(T\right)+\frac{\hbar^{2}}{m}B^{\left(1\right)}\left(T\right)+O\left(\frac{\hbar^{4}}{m^{2}}\right),$$

where

(2) $$B^{\left(0\right)}\left(T\right)=\frac{\Omega_{d}}{2}\int_{0}^{\infty }dr\;r^{d-1}\left[1-e^{-\beta \phi (r)}\right],\;\Omega_{d}=\frac{2\pi^{\frac{d}{2}}}{\Gamma (\frac{d}{2})},$$

is the classical contribution, and

(3) $$B^{\left(1\right)}\left(T\right)=\frac{\Omega_{d}\beta^{3}}{24}\int_{0}^{\infty }dr\;r^{d-1}e^{-\beta \phi (r)}{\left[\frac{d\phi (r)}{dr}\right]}^{2}$$

represents the first quantum correction. In Equations (2) and (3), ϕ(r) denotes the pair potential, $\beta \equiv {\left(k_{B}T\right)}^{-1}$, and $\Omega_{d}$ is the total solid angle in *d* dimensions.

The prototypical pair potential in liquid-state theory is the Lennard-Jones (LJ) potential, written as follows:

(4) $$\phi \left(r\right)=4\epsilon \left[{\left(\frac{\sigma }{r}\right)}^{2n}-{\left(\frac{\sigma }{r}\right)}^{n}\right],$$

where ϵ and σ set the energy and length scales, respectively. The standard LJ (sLJ) fluid corresponds to d=3 and n=6, while the generalized Lennard-Jones (gLJ) model allows arbitrary dimensionality *d* and stiffness parameter n>d. In the limit n→∞, the gLJ potential of Equation (4) approaches the hard-sphere potential.

By introducing the reduced (dimensionless) coefficients, written as follows:

(5) $$B_{c}\equiv \frac{2d}{\Omega_{d}\sigma^{d}}B^{\left(0\right)},\;B_{q}\equiv \frac{24\epsilon }{\Omega_{d}\sigma^{d-2}}B^{\left(1\right)},$$

we obtain

(6a) $$B_{c}\left(T^{*}\right)=d\int_{0}^{\infty }dx\;x^{d-1}\left[1-e^{-4\beta^{*}\left(x^{-2n}-x^{-n}\right)}\right],$$

(6b) $$B_{q}\left(T^{*}\right)=16n^{2}{\beta^{*}}^{3}\int_{0}^{\infty }dx\;x^{-(2n+3-d)}e^{-4\beta^{*}\left(x^{-2n}-x^{-n}\right)}\left(1-4x^{-n}+4x^{-2n}\right),$$

where $T^{*}\equiv 1/\beta^{*}=k_{B}T/\epsilon$ is the reduced temperature.

Several equivalent representations of $B_{c}$ for the sLJ fluid can be found in the literature (see, for instance, Ref. [8] and references therein). Perhaps the most compact expression—valid for the gLJ fluid—is Section 3.7 in [9]:

(7) $$B_{c}\left(T^{*}\right)=\Gamma \left(1-\frac{d}{n}\right){\left(8\beta^{*}\right)}^{\frac{d}{2n}}e^{\beta^{*}/2}D_{\frac{d}{n}}\left(-\sqrt{2\beta^{*}}\right),$$

where we use the integral representation

(8) $$D_{a}\left(z\right)=\frac{e^{-z^{2}/4}}{\Gamma (-a)}\int_{0}^{\infty }dt\;t^{-a-1}\left[e^{-t^{2}/2-zt}-\Theta \left(a\right)\right],\;a<1,$$

for the parabolic cylinder function (Equation (12.5.1) Available online: https://dlmf.nist.gov/12.5.E1 (accessed on 16 November 2025)) [10]. In Equation (8), Θ(a) denotes the Heaviside step function. It ensures convergence of the integral at the lower integration limit. For a<0, the integrand $t^{-a-1}e^{-t^{2}/2-zt}$ is integrable at t=0 without modification. However, for 0<a<1, the behavior $t^{-a-1}$ near t=0 causes a divergence. The subtraction of Θ(a)=1 removes the leading constant term from the small-*t* expansion of the exponential, regularizing the integral while preserving the correct value of $D_{a}\left(z\right)$ through analytic continuation in *a*.

Naturally, the situation is more involved for the quantum contribution $B_{q}$. In a recent work, Zhao et al. [8] derived a linear, second-order homogeneous ordinary differential equation for the sLJ coefficient $B_{q}$. From its solution, they obtained the following:

(9) $$B_{q}\left(T^{*}\right)=\frac{1}{3\times 2^{\frac{11}{6}}}\left[\Gamma \left(\frac{5}{12}\right)F\left(T^{*}\right)-\Gamma \left(-\frac{1}{12}\right)G\left(T^{*}\right)\right],$$

where

(10a) $$F\left(T^{*}\right)={\beta^{*}}^{\frac{19}{12}}\left[72_{1}F_{1}\left(\frac{5}{12};\frac{1}{2};\beta^{*}\right)-22_{1}F_{1}\left(\frac{5}{12};\frac{3}{2};\beta^{*}\right)\right],$$

(10b) $$G\left(T^{*}\right)={\beta^{*}}^{\frac{13}{12}}\left[12{\beta^{*}}_{1}F_{1}\left(\frac{11}{12};\frac{3}{2};\beta^{*}\right)+11_{1}F_{1}\left(-\frac{1}{12};\frac{1}{2};\beta^{*}\right)\right].$$

Here, F11a;b;z denotes the Kummer confluent hypergeometric function (Equation (13.2.2) Available online: https://dlmf.nist.gov/13.2.E2 (accessed on 16 November 2025)) [10]. The result given by Equations (9) and (10) was first obtained by Michels [4].

## 2. First Quantum Correction to the Second Virial Coefficient

Our goal is to derive an alternative, more compact expression for $B_{q}$ in the broader case of the gLJ fluid. We begin by stating the final result:

(11) $$B_{q}\left(T^{*}\right)=n\frac{\Gamma (2-\frac{d-2}{n})}{8}{\left(8\beta^{*}\right)}^{\frac{d-2}{2n}+1}e^{\beta^{*}/2}\left[D_{\frac{d-2}{n}}\left(-\sqrt{2\beta^{*}}\right)+D_{\frac{d-2}{n}-2}\left(-\sqrt{2\beta^{*}}\right)\right].$$

Before proving Equation (11), we list several useful properties of the parabolic cylinder function [10]:

(12a) $$D_{a}\left(z\right)=zD_{a-1}\left(z\right)+\left(1-a\right)D_{a-2}\left(z\right),$$

(12b) $$\frac{\partial D_{a}\left(z\right)}{\partial z}=aD_{a-1}\left(z\right)-\frac{z}{2}D_{a}\left(z\right),$$

(12c) $$\lim_{z\to 0}D_{a}\left(z\right)=\frac{\sqrt{\pi }2^{\frac{a}{2}}}{\Gamma (\frac{1-a}{2})},\;\lim_{z\to \infty }D_{a}\left(-z\right)=\frac{\sqrt{2\pi }}{\Gamma (-a)}e^{z^{2}/4}z^{-a-1},$$

(12d) $$D_{0}\left(z\right)=e^{-z^{2}/4},\;D_{-2}\left(z\right)=e^{-z^{2}/4}-\sqrt{\frac{\pi }{2}}e^{z^{2}/4}z\;\mathrm{erfc}\left(\frac{z}{\sqrt{2}}\right),$$

(12e) $$D_{a}\left(\sqrt{2}z\right)=2^{-\frac{a}{2}}e^{-z^{2}/2}H_{a}\left(z\right).$$

Equation (12e) defines the generalized Hermite functions $H_{a}\left(z\right)$ for arbitrary (noninteger) degree a<1 (Equation (12.7.2) Available online: https://dlmf.nist.gov/12.7.E2 (accessed on 16 November 2025)) [10].

By introducing the change of variable $x\to t=\sqrt{8\beta^{*}}x^{-n}$ in Equation (6b), we obtain the following:

(13) $$B_{q}\left(T^{*}\right)=\frac{n}{8}{\left(8\beta^{*}\right)}^{\frac{a}{2}+1}\int_{0}^{\infty }dt\;t^{-a+1}e^{-t^{2}/2-zt}\left(z^{2}+2zt+t^{2}\right),$$

where we have used the notation $a\equiv \frac{d-2}{n}$, $z\equiv -\sqrt{2\beta^{*}}$. Using the integral representation of the parabolic cylinder function [see Equation (8)], Equation (13) can be rewritten as follows:

(14) $$\begin{array}{cc} B_{q}\left(T^{*}\right)= & n\frac{\Gamma (2-a)}{8}{\left(8\beta^{*}\right)}^{\frac{a}{2}+1}e^{z^{2}/4}[z^{2}D_{a-2}\left(z\right)+2\left(2-a\right)zD_{a-3}\left(z\right)\\ & +\left(3-a\right)\left(2-a\right)D_{a-4}\left(z\right)]. \end{array}$$

This expression is already quite compact, but it can be further simplified. Iterative application of Equation (12a) yields the following:

(15) $$\begin{array}{cc} D_{a}\left(z\right) & =z\left[zD_{a-2}\left(z\right)+\left(2-a\right)D_{a-3}\left(z\right)\right]+\left(1-a\right)D_{a-2}\left(z\right)\\ & =z^{2}D_{a-2}\left(z\right)+\left(2-a\right)zD_{a-3}\left(z\right)+\left(2-a\right)D_{a-2}\left(z\right)-D_{a-2}\left(z\right). \end{array}$$

Next, we apply Equation (12a) to the term $\left(2-a\right)D_{a-2}\left(z\right)$, which gives the following:

(16) $$D_{a}\left(z\right)=z^{2}D_{a-2}\left(z\right)+2\left(2-a\right)zD_{a-3}\left(z\right)+\left(3-a\right)\left(2-a\right)D_{a-4}\left(z\right)-D_{a-2}\left(z\right).$$

Substituting this identity into Equation (14), and returning to the physical variables $a\to \frac{d-2}{n}$ and $z\to -\sqrt{2\beta^{*}}$, we recover Equation (11). In terms of the generalized Hermite functions, Equation (11) can be rewritten as follows:

(17) $$B_{q}\left(T^{*}\right)=n\frac{\Gamma (2-\frac{d-2}{n})}{4}{\left(4\beta^{*}\right)}^{\frac{d-2}{2n}+1}\left[H_{\frac{d-2}{n}}\left(-\sqrt{\beta^{*}}\right)+2H_{\frac{d-2}{n}-2}\left(-\sqrt{\beta^{*}}\right)\right].$$

For the particular case of the sLJ model (d=3, n=6), the following is written:

(18) $$\begin{array}{cc} B_{q}\left(T^{*}\right) & =\frac{3\Gamma (\frac{11}{6})}{4}{\left(8\beta^{*}\right)}^{\frac{13}{12}}e^{\beta^{*}/2}\left[D_{\frac{1}{6}}\left(-\sqrt{2\beta^{*}}\right)+D_{-\frac{11}{6}}\left(-\sqrt{2\beta^{*}}\right)\right]\\ & =\frac{3\Gamma (\frac{11}{6})}{2}{\left(4\beta^{*}\right)}^{\frac{13}{12}}\left[H_{\frac{1}{6}}\left(-\sqrt{\beta^{*}}\right)+2H_{-\frac{11}{6}}\left(-\sqrt{\beta^{*}}\right)\right]. \end{array}$$

It can be verified that Equation (18) is equivalent to the combination of Equations (9) and (10).

The limits given by Equation (12c) allow us to determine the low- and high-temperature behaviors of $B_{q}\left(T^{*}\right)$ for the gLJ fluid:

(19) $$\lim_{T^{*}\to 0}B_{q}\left(T^{*}\right)=n\sqrt{\pi }2^{\frac{d-2}{n}+1}e^{1/T^{*}}{T^{*}}^{-\frac{3}{2}},\;\lim_{T^{*}\to \infty }B_{q}\left(T^{*}\right)=n\frac{\Gamma (2-\frac{d-2}{2n})}{2}{\left(\frac{4}{T^{*}}\right)}^{\frac{d-2}{2n}+1}.$$

In the second equality of Equation (19), use has been made of the identity $\Gamma \left(2x\right)/\Gamma \left(x\right)=2^{2x-1}\Gamma \left(x+\frac{1}{2}\right)/\sqrt{\pi }$.

Interestingly, Equation (11) simplifies considerably in the case of a two-dimensional fluid (d=2). Using Equation (12d), we obtain the following:

(20) $$B_{q}\left(T^{*}\right)=n\beta^{*}\left[2+\sqrt{\pi \beta^{*}}e^{\beta^{*}}\;\mathrm{erfc}\left(-\sqrt{\beta^{*}}\right)\right].$$

In this case, the ratio $B_{q}/n$ is independent of the stiffness parameter *n*. It is worth mentioning that Equation (20) also provides the ratio $B_{q}/n$ in the limit n→∞ for any dimensionality.

**Figure 1:**
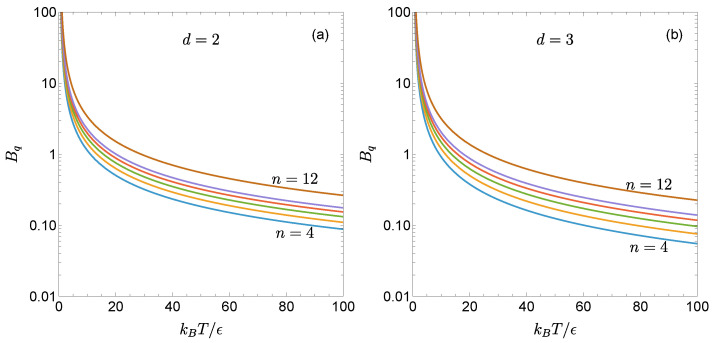
Reduced first quantum correction to the second virial coefficient, as given by Equation (11), for the gLJ fluid with (**a**) d=2 and (**b**) d=3. The curves correspond, from bottom to top, to stiffness parameters n=4, 5, 6, 7, 8, and 12.

Figure 1 illustrates the temperature dependence of $B_{q}\left(T^{*}\right)$ for the two- and three-dimensional gLJ fluids with n=4, 5, 6, 7, 8, and 12. As can be seen, for a given $T^{*}$, the reduced first quantum correction $B_{q}$ increases as the potential becomes stiffer. Moreover, the influence of *n* is more pronounced at high temperatures than at low temperatures, consistent with the limiting behaviors described by Equation (19). For the same values of $T^{*}$ and *n*, $B_{q}$ is larger in the two dimensions than in three.

## 3. First Quantum Correction to the Boyle Temperature

From Equation (1), the reduced second virial coefficient of the gLJ fluid can be written as follows:

(21) $$\frac{2d}{\Omega_{d}\sigma^{d}}B_{2}\left(T^{*}\right)=B_{c}\left(T^{*}\right)+\frac{d}{12}qB_{q}\left(T^{*}\right)+O\left(q^{2}\right),$$

where the dimensionless quantum parameter

(22) $$q\equiv \frac{\hbar^{2}}{m\sigma^{2}\epsilon }$$

measures the expected magnitude of quantum effects.

The Boyle temperature, $T_{B}^{*}$, is defined by the condition $B_{2}\left(T_{B}^{*}\right)=0$. It marks the balance between the attractive and repulsive contributions to the intermolecular potential: the attractive interactions dominate for $T^{*}<T_{B}^{*}$, whereas the repulsive ones dominate for $T^{*}>T_{B}^{*}$. In the semiclassical regime, the Boyle temperature can be expanded as follows:

(23) $$T_{B}^{*}=T_{0}^{*}-qT_{1}^{*}+O\left(q^{2}\right),$$

where $T_{0}^{*}$ is the classical Boyle temperature, i.e., the solution of $B_{c}\left(T_{0}^{*}\right)=0$, or equivalently, $D_{d/n}\left(-\sqrt{2/T_{0}^{*}}\right)=0$. By inserting Equation (23) into Equation (21), one obtains the first quantum correction to the Boyle temperature,

(24) $$T_{1}^{*}=\frac{d}{12}\frac{B_{q}\left(T_{0}^{*}\right)}{{\left.\partial B_{c}\left(T^{*}\right)/\partial T^{*}\right|}_{T_{0}^{*}}}.$$

Note that $\partial B_{c}/\partial T^{*}=-{T^{*}}^{-2}\partial B_{c}/\partial \beta^{*}$, where, from Equations (7) and (12b), one finds

(25) $$\frac{\partial B_{c}}{\partial \beta^{*}}=\frac{d}{n}\left[\frac{B_{c}}{2\beta^{*}}-\frac{\Gamma (1-\frac{d}{n})}{\sqrt{2\beta^{*}}}{\left(8\beta^{*}\right)}^{\frac{d}{2n}}e^{\beta^{*}/2}D_{\frac{d}{n}-1}\left(-\sqrt{2\beta^{*}}\right)\right].$$

Thus, one finally obtains the following:

(26) $$T_{1}^{*}=n^{2}\frac{\Gamma (2-\frac{d-2}{n})}{3\Gamma (1-\frac{d}{n})}{\left(\frac{T_{0}^{*}}{8}\right)}^{\frac{1}{2}+\frac{1}{n}}\frac{D_{\frac{d-2}{n}}\left(-\sqrt{2/T_{0}^{*}}\right)+D_{\frac{d-2}{n}-2}\left(-\sqrt{2/T_{0}^{*}}\right)}{D_{\frac{d}{n}-1}\left(-\sqrt{2/T_{0}^{*}}\right)}.$$

**Figure 2:**
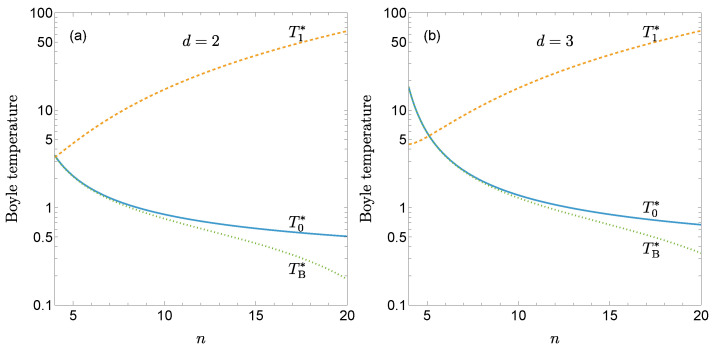
Classical Boyle temperature $T_{0}^{*}$ (solid lines) and its first quantum correction $T_{1}^{*}$ (dashed lines) as functions of the stiffness parameter *n* for the gLJ fluid with (**a**) d=2 and (**b**) d=3. The dotted lines represent the quantum-corrected Boyle temperature $T_{B}^{*}$, obtained from Equation (23) with $q=5\times 10^{-3}$.

Figure 2 shows $T_{0}^{*}$ and $T_{1}^{*}$ as functions of *n* for d=2 and d=3. While the classical Boyle temperature $T_{0}^{*}$ decreases as the potential becomes stiffer, the first quantum correction $T_{1}^{*}$ increases with *n*. As a result, quantum effects amplify the decrease of the Boyle temperature with increasing stiffness, as illustrated by the curves representing $T_{0}^{*}-qT_{1}^{*}$ with $q=5\times 10^{-3}$. This effect is more pronounced in two-dimensional fluids than in three-dimensional ones.

## 4. Application to Noble Gases

In the case of the sLJ model, the influence of quantum effects on the second virial coefficient can be assessed through the relative deviation, written as follows:

(27) $$\begin{array}{cc} \delta B_{2}^{*}\left(T\right) & \equiv \frac{B_{2}\left(T\right)-B^{\left(0\right)}\left(T\right)}{B_{2}\left(T\right)}=\frac{q}{4}\frac{B_{q}\left(T^{*}\right)}{B_{c}\left(T^{*}\right)}+O\left(q^{2}\right)\\ & =q\frac{5\Gamma \left(\frac{5}{6}\right)}{32\sqrt{\pi }}{\left(8\beta^{*}\right)}^{\frac{5}{6}}\frac{D_{\frac{1}{6}}\left(-\sqrt{2\beta^{*}}\right)+D_{-\frac{11}{6}}\left(-\sqrt{2\beta^{*}}\right)}{D_{\frac{1}{2}}\left(-\sqrt{2\beta^{*}}\right)}+O\left(q^{2}\right). \end{array}$$

To first order in the quantum parameter *q*, we note that the relative deviation $\delta B_{2}^{*}\left(T\right)$ is compactly expressed in terms of the parabolic cylinder functions $D_{\frac{1}{2}}$, $D_{\frac{1}{6}}$, and $D_{-\frac{11}{6}}$.

As a simple application, we consider the noble gases helium (He), neon (Ne), and argon (Ar), which can be described by the sLJ potential with the parameter values for ϵ and σ displayed in Table 1 [11]. The atomic masses, *m*, and the dimensionless quantum parameter, *q*, defined in Equation (21) are also included in Table 1.

**Figure 3:**
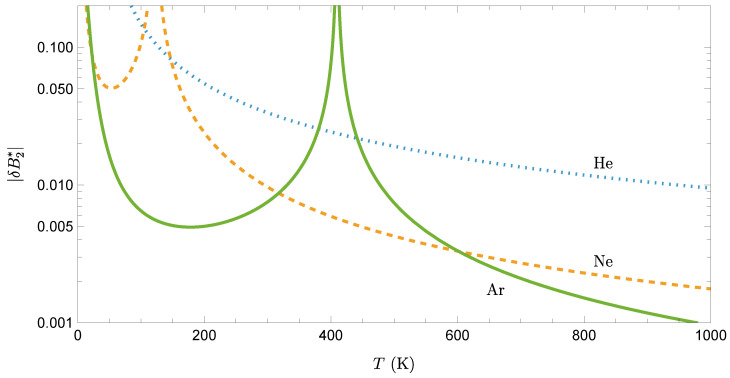
Absolute value of the relative deviation, $|\delta B_{2}^{*}\left(T\right)|$, for helium, neon, and argon, obtained from Equation (27) to first order in *q*.

When comparing $\delta B_{2}^{*}$ for helium, neon, and argon, one finds that two competing effects are at play. On the one hand, since $\epsilon_{\mathrm{He}}<\epsilon_{\mathrm{Ne}}<\epsilon_{\mathrm{Ar}}$, a common fixed temperature *T* corresponds to $T_{\mathrm{He}}^{*}≃11.7\;T_{\mathrm{Ar}}^{*}>T_{\mathrm{Ne}}^{*}≃3.36\;T_{\mathrm{Ar}}^{*}>T_{\mathrm{Ar}}^{*}$, so that, in view of Figure 1b for n=6, $B_{q,\mathrm{He}}<B_{q,\mathrm{Ne}}<B_{q,\mathrm{Ar}}$. On the other hand, $q_{\mathrm{He}}≃204.4\;q_{\mathrm{Ar}}>q_{\mathrm{Ne}}≃10.2\;q_{\mathrm{Ar}}>q_{\mathrm{Ar}}$. The second effect dominates, as can be observed in Figure 3.

Note that, because $B_{c}\left(T^{*}\right)$ vanishes at the classical Boyle temperature $T_{0}^{*}≃3.42$, $|\delta B_{2}^{*}\left(T\right)|$ diverges at $T=T_{0}^{*}\epsilon /k_{B}$, as seen in Figure 3 for neon and argon. Apart from this singularity, since quantum effects scale roughly as ${\left(m\epsilon \right)}^{-1}$, helium exhibits the strongest quantum corrections, neon has moderate corrections, and argon has the weakest quantum effects at any given temperature. This behavior explains why helium remains liquid at atmospheric pressure down to absolute zero (quantum effects prevent solidification), while argon solidifies at 84 K and behaves largely classically at typical temperatures.

## 5. Outlook

Although in this work we have focused on the first quantum correction to $B_{2}$, the same methodology can be extended to higher-order terms. The convergence of the quantum expansion in powers of ${¯\;h}^{2}/m$ depends on the temperature regime and the strength of the interaction. For systems like helium at very low temperatures, higher-order corrections may become necessary for quantitative accuracy.

In particular, the general expression for the second-order correction reads [1,2] as follows:

(28) $$\begin{array}{cc} B^{\left(2\right)}\left(T\right)= & -\frac{\Omega_{d}\beta^{4}}{24}\int_{0}^{\infty }dr\;r^{d-1}e^{-\beta \phi (r)}\left\{\frac{1}{10}{\left[\frac{d^{2}\phi \left(r\right)}{dr^{2}}\right]}^{2}+\frac{1}{5r^{2}}{\left[\frac{d\phi (r)}{dr}\right]}^{2}\right.\\ & \left.+\frac{\beta }{9r}{\left[\frac{d\phi (r)}{dr}\right]}^{3}-\frac{\beta^{2}}{72}{\left[\frac{d\phi (r)}{dr}\right]}^{4}\right\}. \end{array}$$

Specializing to the gLJ potential, Equation (4), and introducing the change of variable $r\to t=\sqrt{8\beta^{*}}{\left(r/\sigma \right)}^{-n}$, one can express $B^{\left(2\right)}$ in terms of the parabolic cylinder functions $D_{a-2}\left(-\sqrt{2\beta^{*}}\right)$, $D_{a-3}\left(-\sqrt{2\beta^{*}}\right)$, …, $D_{a-8}\left(-\sqrt{2\beta^{*}}\right)$, with $a=\frac{d-4}{n}$. This expression can be further simplified through repeated application of Equation (12a).

The systematic nature of our approach—reducing complex integrals to combinations of parabolic cylinder functions—extends naturally to arbitrary orders. This provides a practical computational framework for exploring the convergence properties of the quantum expansion and for determining when higher-order terms become significant. Such analysis would be particularly relevant for light atoms like helium at temperatures below ∼50 K, where the ratio $qB_{q}\left(T^{*}\right)/4B_{c}\left(T^{*}\right)$ approaches unity and second-order corrections are no longer negligible.

## 6. Conclusions

In this paper, we have derived an explicit and compact expression, Equation (11), for the first quantum correction to the second virial coefficient of a *d*-dimensional fluid composed of particles interacting through the gLJ (2n,n) potential defined in Equation (4). As in the classical case, Equation (8), the first quantum correction has been conveniently expressed in terms of parabolic cylinder functions. For the particular case of the sLJ fluid (d=3, n=6), the expression obtained here for $B_{q}$ [see Equation (18)] is considerably more concise than the combination of Equations (9) and (10) reported previously [4,8].

An additional asset of the present results is that they allow one to explore the combined influence of dimensionality and stiffness on the quantum correction $B_{q}$. From Equation (11), it follows that the ratio $B_{q}/n$ depends on *d* and *n* only through the combination (d−2)/n. This implies that, at a given reduced temperature $T^{*}$, the value of $B_{q}/n$ for a *d*-dimensional fluid (d>3) with stiffness *n* is identical to that of a three-dimensional fluid with an effective stiffness $n_{\mathrm{eff}}=n/\left(d-2\right)$. In contrast, for two-dimensional fluids, $B_{q}/n$ is independent of *n* and is given by the particularly simple expression of Equation (20), which is also applicable to any *d* in the limit n→∞.

The knowledge of $B_{q}$ has enabled us to derive the first quantum correction to the Boyle temperature [see Equation (26)]. As illustrated by Figure 1 and Figure 2, the general trend is that the quantum corrections to both the second virial coefficient and the Boyle temperature become more significant as the potential stiffness increases and the system dimensionality decreases.

We have applied our results to the noble gases helium, neon, and argon, demonstrating that the relative quantum correction $\delta B_{2}^{*}\left(T\right)$ decreases significantly (in absolute value) from helium to argon, primarily due to the strong dependence on the quantum parameter $q\propto {\left(m\epsilon \right)}^{-1}$. This application illustrates the practical utility of our compact expressions for assessing quantum effects in real physical systems.

In summary, we have obtained a compact and fully explicit expression for the first quantum correction to the second virial coefficient of a *d*-dimensional gLJ fluid, expressed in terms of parabolic cylinder or generalized Hermite functions. The formulation unifies the treatment of dimensionality and stiffness, provides analytic access to the limiting behaviors, and naturally yields the quantum correction to the Boyle temperature. Beyond its intrinsic theoretical interest, the approach presented here provides a systematic framework for deriving higher-order quantum corrections (as discussed in Section 5) of relevance in quantum and semiclassical fluid theory, and its application to noble gases demonstrates its utility for understanding quantum effects in real molecular systems. The compact analytical nature of our results also makes them particularly valuable for pedagogical purposes, providing students and researchers with transparent expressions that reveal the underlying structure of quantum corrections and facilitate the development of physical intuition about quantum effects in fluids.

## Figures and Tables

**Table 1:** Values of *m*, ϵ, σ, and *q* for the noble gases He, Ne, and Ar.

Gas	*m* (kg)	$\epsilon /k_{B}$ (K)	σ (m)	*q*
He	$6.646\times 10^{-27}$	10.22	$2.576\times 10^{-10}$	0.179
Ne	$3.351\times 10^{-26}$	35.70	$2.749\times 10^{-10}$	$8.91\times 10^{-3}$
Ar	$6.634\times 10^{-26}$	119.8	$3.405\times 10^{-10}$	$8.74\times 10^{-4}$

## Data Availability

The raw data supporting the conclusions of this article will be made available by the authors on request.

## Abbreviations

The following abbreviations are used in this manuscript:

gLJ	generalized Lennard-Jones
LJ	Lennard-Jones
sLJ	standard Lennard-Jones
